# Supplementary material for: Bystander intervention is associated with reduced early mortality among injury victims in Cameroon
Source: PLOS Glob Public Health. 2024 Jul 11;4(7):e0002875. doi: 10.1371/journal.pgph.0002875 (PMC11238979; doi:10.1371/journal.pgph.0002875)
Supplement: Attachment — Submitted filename: PLOS Response to Reviewers.docx [file pgph.0002875.s002.docx]

Dr. Uzma Rahim Khan

Academic Editor

PLOS Global Public Health

20 February 2024

Dear Dr. Khan,

Subject: Submission of revised paper PGPH-D-24-00069 “Bystander intervention is associated with reduced mortality among injury victims in Cameroon”

Thank you for your email on February 19^th^ detailing the reviewers’ comments. We carefully reviewed the comments and have edited the manuscript to reflect them. Changes to the manuscript are reflected in red via track changes. Specifically, we addressed each point:

1. *“Please compare results with existing literature”* Reviewer 1

We added information in the discussion about existing literature about layperson first responder programs.

2. *“Study setting elaborates the broad description of setting that is giving you study population, hence remains unclear here.” “Describe the study setting for the reader to understand the context. (City/village/state/province).”* Reviewer 2

In the “study setting” section, we added information about the utilization of ambulances in Cameroon. Otherwise, little is known about the landscape of prehospital care in Cameroon prior to this study.

3. *“Overview of the hospitals: How many hospitals? Type of hospitals (public, private)? (tertiary, secondary)? Patient population catered to (urban/rural)?*
4. *Briefly explain the trauma registry methodology to better understand the data collection. Who collected the data? Where did they collect the data (Emergency department, wards)? Was data extracted from medical records as well? Data collection timing/duration? Was the data collected digitally or paper based? Who calculated the Injury Severity Score?”* Reviewer 2

In the “Cameroon Trauma Registry” section, we added information about how data is collected by research assistants and recorded as well as calculation of injury severity. We also added some context about the hospitals included in the registry, including their urban-rural and public-private status.

5. *“Lines 157-159 are repeated.”* Reviewer 2

Corrected.

6. *“Pre-hospital care: how was the medical information for prehospital care collected? Who gave history? Driver, transporters, bystanders may not witness the entire scene?”* Reviewer 2

We inserted discussion in the methods about who provides history of prehospital care provided, if any, when a patient presents to the hospital. These are the patient themselves, transporters, and witnesses.

7. *“Pre-hospital care: What was the first referral facility? Were all these patients brought directly to the hospitals included in the study or were they first taken elsewhere and then brought later after stabilization?”* Reviewer 2

Patients were not excluded if they presented to another facility or formal medical care prior to presentation, but this was not considered prehospital care. This was added to the methods section.

8. *“It would be interesting to see the multivariate results adjusted for injury type, mode of transportation, first responder.”* Reviewer 2

Given that death was rare among trauma patients (4.5% of cohort), multivariate analysis would not support these additional variables. When looking at the correlation of these mentioned variables on univariate analysis, it is true that injury type is independently associated with survival. Specifically, penetrating and blunt injury types are associated with reduced survival; however, this variable is colinear with injury severity as was already included in multivariate analysis. Type of responder was not independently associated with survival thus was not included in the analysis. Finally, mode of transportation did have significant pertinence for survival on univariate analysis (p<0.001); specifically, 35% of patients transported by police died. This was not included in the analysis because this is confounded by that just 4.3% of those transported by police received prehospital care compared to 21% of the entire cohort.

9. *“The authors need to discuss their results and findings especially focusing on the bystanders.”* Reviewer 2

The way that data is collected in the trauma registry, bystander is a separate category from other untrained people who were possibly proximate witnesses to a trauma like a person involved or friend. We chose to consider those relatives or person involved as untrained responders. Under this definition of “untrained responder,” 98% of the prehospital care provided falls within this category.

10. *“The % for PC and NPC are different in abstract (line 72) and results section (line 199)”* Reviewer 2

In the abstract, it is referring to all patients. In the Table 1, it refers to just the male cohort to demonstrate that there was no difference in prehospital care provision between male and female patients.

We hope the revised version is now suitable for publication. Thank you for your consideration. We look forward to hearing from you soon.

Sincerely,

Kathleen O’Connor

University of Pittsburgh School of Medicine
