# Supplementary material for: Bystander intervention is associated with reduced early mortality among injury victims in Cameroon
Source: PLOS Glob Public Health. 2024 Jul 11;4(7):e0002875. doi: 10.1371/journal.pgph.0002875 (PMC11238979; doi:10.1371/journal.pgph.0002875)
Supplement: Attachment — Submitted filename: PLOS Response to Reviewers 3.docx [file pgph.0002875.s003.docx]

Dr. Uzma Rahim Khan

Academic Editor

PLOS Global Public Health

9 April 2024

Dear Dr. Khan,

Subject: Submission of revised paper PGPH-D-24-00069 “Bystander intervention is associated with reduced early mortality among injury victims in Cameroon”

Thank you for your email on March 27^th^ detailing the reviewers’ comments. We have carefully reviewed the comments and have revised the manuscript accordingly. Accompanying this letter, please see the revised draft with changes to the manuscript reflected in red and blue via track changes.

Specifically:

1. *“This topic and information is of great importance in field of trauma and injury. However, the title the aim of study and conclusion are loosing consistency and coherence. Clear study objective statement needed to be mentioned for clarity.”*

We would like to thank the reviewer for their comment and this opportunity to strengthen our manuscript. We have streamlined and reworded sections of our introduction to improve consistency and coherency. We have more clearly stated the specific objective of the study.

2. *Revisions have been made still discussions needs improvement in terms of reflection/reasoning and evidence relevance."Bystanders pre hospital intervention" This essence is lost from discussion till conclusion. It is very important and innovative to highlight bystanders intervention and investment in this area in terms of capacity building /raising awareness or life skills/first aid.*

We would like to thank the reviewer for their comment and this opportunity to strengthen our manuscript. We have re-framed our discussion and conclusion to more coherently and impactfully summarize the study findings. We have emphasized the association between bystander intervention and improved survival and more thoroughly discussed the implications of this finding in view of other literature and a call for capacity building in both Cameroon and other LMIC. Finally, we added “early” to the title to better describe the impact of bystander intervention on mortality.

In addition to the above changes, we added citations to better support our treatment of lay first responder programs in the discussion section, inserted an acknowledgement of our collaborators, and made grammatical corrections. We hope that you find this revised version of sufficient interest and impact to merit publication. On behalf of our collaboration, we greatly appreciate your ongoing consideration of our work. Please do not hesitate to contact us at any time for any questions or additional revisions.

Sincerely,

Kathleen O’Connor

University of Pittsburgh School of Medicine
