# Supplementary material for: Bystander intervention is associated with reduced early mortality among injury victims in Cameroon
Source: PLOS Glob Public Health. 2024 Jul 11;4(7):e0002875. doi: 10.1371/journal.pgph.0002875 (PMC11238979; doi:10.1371/journal.pgph.0002875)
Supplement: Attachment — Submitted filename: PLOS Response to Reviewers 4.docx [file pgph.0002875.s004.docx]

Barnabas Tobi Alayande

Academic Editor

PLOS Global Public Health	18 June 2024

Dear Dr. Alayande,

Subject: Submission of revised paper PGPH-D-24-00069 “Bystander intervention is associated with reduced early mortality among injury victims in Cameroon”

Thank you for your email on June 4^th^ detailing the reviewers’ comments. We have carefully reviewed the comments and have revised the manuscript accordingly. Accompanying this letter, please see the revised draft with changes to the manuscript reflected in red and blue via track changes.

Specifically:

1. *It is good practice to include the ethics approval numbers e.g (2018/09/1094/CE/CNERSH/SP for Le Comite National d’Ethique de la Recherche pour la Sante Humaine (CNERSH), and the University of California Los Angeles (IRB#19-000086) etc. in the body of the manuscript within the Ethics section for clarity.*

Ethic approval numbers added to the methods.

2. *Header rows of the tables should be written in full (even if they fit 2 lines each) so that they are stand-alone in a sense; as NPC, PC etc are not clear within the table (even if this has been described within the text). A superscript on the abbreviations on the table connected to a footer where these abbreviations are written in full is also an option.*

Full titles added to header rows.

3. *As first, second, corresponding, and positionally last author (co-senior authorship acknowledged) are from the global north on this Cameroon focused project, it will be advisable for the authors to submit a reflexivity statement to show how they developed local junior researcher capacity by this study, and perhaps how they hope to include more junior researchers in leading writing subsequently, in the spirit of global health research equity promoted by Plos Global Public Health.*

We agree that it is a priority for us to build research capacity among African junior scientists. We have specified how they will continue to play key roles in our future work in a reflexivity statement contained in the methods section.

We hope that you find this revised version of sufficient interest and impact to merit publication. On behalf of our collaboration, we greatly appreciate your ongoing consideration of our work. Please do not hesitate to contact us at any time for any questions or additional revisions.

Sincerely,

Kathleen O’Connor

University of Pittsburgh School of Medicine
